# Racial effects on Masimo pulse oximetry: a laboratory study

**DOI:** 10.1007/s10877-022-00927-w

**Published:** 2022-11-12

**Authors:** Steven J. Barker, William C. Wilson

**Affiliations:** 1 Chief Science Officer, Masimo Corp, Irvine, CA USA; 2 Chief Medical Officer and Senior Vice President of Clinical Research and Medical Affairs, Masimo Corp, Irvine, CA USA

**Keywords:** Pulse oximetry, Oxygen saturation, Race, Ethnicity, Skin pigmentation, Occult hypoxemia, Masimo

## Abstract

Recent publications have suggested that pulse oximeters exhibit reduced accuracy in dark-skinned patients during periods of hypoxemia. Masimo SET® (Signal Extraction Technology®) has been designed, calibrated, and validated using nearly equal numbers of dark and light skinned subjects, with the goal of eliminating differences between pulse oximetry saturation (SpO_2_) and arterial oxygen saturation (SaO_2_) values due to skin pigmentation. The accuracy concerns reported in dark-skinned patients led us to perform a retrospective analysis of healthy Black and White volunteers. Seventy-five subjects who self-identified as being racially Black or White underwent a desaturation protocol where SaO_2_ values were decreased from 100 to 70%, while simultaneous SpO_2_ values were recorded using Masimo RD SET® sensors. Statistical bias (mean difference) and precision (standard deviation of difference) were − 0.20 ± 1.40% for Black and − 0.05 ± 1.35% for White subjects. Plots of SpO_2_ versus SaO_2_ show no significant visible differences between races throughout the saturation range from 70 to 100%. Box plots grouped in 1% saturation bins, from 89–96%, and plotted against concomitant SaO_2_ values, show that occult hypoxemia (SaO_2_ < 88% when SpO_2_ = 92–96%) occurred in only 0.2% of White subject data pairs, but not in any Black subjects. There were no clinically significant differences in bias (mean difference of SpO_2_-SaO_2_) found between healthy Black and White subjects. Occult hypoxemia was rare and did not occur in Black subjects. Masimo RD SET® can be used with equal assurance in people with dark or light skin. These laboratory results were obtained in well-controlled experimental conditions in healthy volunteers—not reflecting actual clinical conditions/patients.

## Introduction

The effect of skin color on the accuracy of oxygen saturation measured by pulse oximetry (SpO_2_) has been a topic of discussion for many years [1, 2]. The tendency of some pulse oximeters to overestimate arterial oxygen saturation (SaO_2_) in healthy dark-skinned volunteer subjects was first reported by Bickler et al. in 2005 [1]. The same investigators later found that this positive bias (tendency of SpO_2_ to overestimate SaO_2_) was greatest at SaO_2_ values below 80%, suggesting an increased risk of missed hypoxemic events [2].

Several recent publications have raised new questions regarding the accuracy of present-day pulse oximeters in different races. Sjoding et al. performed a retrospective, multi-center study of pulse oximeters in ICU patients [3]. They concluded that, “in two large cohorts, Black patients had nearly three times the frequency of occult hypoxemia that was not detected by pulse oximetry as White patients.” As with nearly all the recent reports, Sjoding et al. pooled data from multiple pulse oximeter manufacturers, generalizing claims on pulse oximetry without respect to manufacturer, model, or sensor type. In a more recent clinical study in COVID-19 patients, Crooks et al. found that in the 85–89% SaO_2_ range, their pulse oximeters overestimated SaO_2_ by an average of 5.8% in Asians, 3.9% in Blacks, and 2.4% in Whites [4]. In another retrospective clinical study, Burnett et al. found that in 151,000 paired readings of SpO_2_ versus SaO_2_, the pulse oximeters showed an incidence of occult hypoxemia (i.e., missed hypoxemic events) of 2.1% in Blacks, 1.8% in Hispanics, and 1.1% in Whites [5]. Recent published studies by Fawzy et al. and by Chesley et al. yielded similar results and conclusions regarding racial differences in occult hypoxemia [6, 7].

Masimo has accounted for the potential confounder of skin pigmentation on pulse oximetry measurements [8], and designed Masimo SET® (Signal Extraction Technology®) using a unique signal-processing method and other engineered solutions to minimize the impact of skin pigmentation and other common pulse oximetry confounders, including motion and low perfusion. In view of the recent concerns regarding pulse oximetry accuracy differences between Black and White patients, we conducted a retrospective laboratory evaluation to assess the accuracy of Masimo SET® pulse oximetry with RD SET® sensors on healthy Black and White volunteers undergoing controlled desaturation studies with SaO_2_ values ranging between 100 and 70%.

## Methods

Human volunteer desaturation data collected in the Masimo laboratory between September 2015 and July 2021 were retrospectively evaluated. The protocol underwent review and approval was granted by the Institutional Review Board of Ethical & Independent (E&I) Review Services (Lee’s Summit, MO). Written informed consent was obtained from each subject prior to enrollment. All subjects met inclusion criteria to participate, including a thorough health history screening assessment specifically targeting any systemic diseases or current health conditions that could increase study risk. A complete list of exclusion criteria, including screened health conditions, is provided in Appendix 1. Consequently, enrolled subjects were classified as either ASA I ("a normal healthy subject") or ASA II ("a subject with mild systemic disease") on the ASA Physical Status Classification System. Some subjects were involved in previous studies involving healthy volunteers in the Masimo laboratory, which were not related to SpO_2_. A few subjects were also involved in previous SpO_2_ studies, not related to this study, and those data points were not included in this dataset. None of the subjects in this study participated in prior Masimo SET® calibration studies, and none of the prior studies have been published.

The data include 7183 paired samples (3201 Black and 3982 White) obtained from 75 subjects (39 Black and 36 White) who self-identified as being either racially Black or White. With 75 subjects measured, there were total 87 visits, with five subjects in each group having two or three repeat visits. Since there were long intervals between the repeat visits, the study data from multiple visits was considered independent, and none of the visits were excluded from analysis. Demographic data are summarized in Table 1, including: age, sex, race, baseline total hemoglobin (tHb), Carboxyhemoglobin (COHb), Methemoglobin (MetHb), and Massey Scale, a validated index of skin tone [9].

**Table 1:** Demographic data for Black and White subjects, as well as combined data

Masimo SET	N	Paired Samples	Age* mean (range)	Sex	Massey Scale median (range)	tHb (g/dL) mean (range)	COHb % Mean (range)	MetHb % Mean (range)
Black	39	3201	34.8 (21–50)	25 M, 14F	6 (4–9)	14.5 (11.3–18.2)	1.1 (0.3–1.8)	1.1 (0.3–1.6)
White	36	3982	30.4 (18–44)	23 M, 13F	2 (1–4)	14.5 (11.2–18.1)	0.9 (0.3–1.6)	1.1 (0.3–1.5)
All	75	7183	32.7 (18–50)	48 M, 27F	4 (1–9)	14.5 (11.2–18.2)	1.0 (0.3–1.8)	1.1 (0.3–1.6)

*Age expressed in years, *tHb—*total hemoglobin, *COHb—*Carboxyhemoglobin, *MetHb*—Methemoglobin

**Fig. 1:**
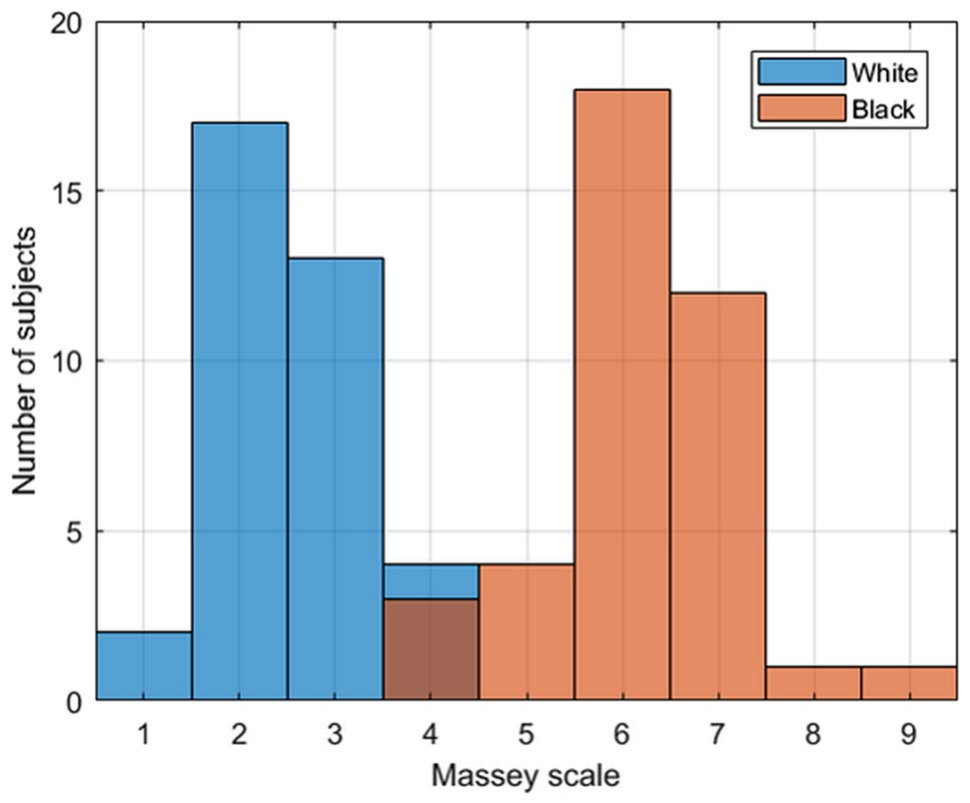
Massey Scale skin color versus number of subjects for self-identified White (blue tint boxes) and Black (salmon tint boxes) subjects

Subjects who self-identified as Black had Massey Scale values ranging from 4–9, while those who self-identified as White had Massey Scale values of 1–4. The Massey Scale values are shown in Table 1, and graphically in Fig. 1.

The SpO_2_ values obtained from Masimo SET® pulse oximeters (MX technology boards) with RD-SET® sensors (Masimo, Irvine, California) were time-matched to be recorded simultaneously with the arterial blood gas (ABG) samples obtained from a radial arterial cannula, and analyzed on a Radiometer ABL-835 Flex CO-Oximeter (Radiometer Inc., Brea, California), which was calibrated daily before use. The ABG samples were collected and handled in accordance with the guidelines provided by the blood gas analyzer manufacturer [10, 11].

The subjects were exposed to a desaturation protocol that sequentially decreased the SpO_2_ in a stepwise fashion, achieving six stable plateau values between 100 and 70%, while recording simultaneous SaO_2_ readings. Multiple replicates were obtained from subjects at each plateau depending on stability of the SpO2 reference, and subject safety and comfort. The protocol was consistent with the ISO 80601–2-61 pulse oximetry standard. Data were grouped by self-declared race (Black vs White) and analyzed separately for each group. There was a median of 72 samples per subject for the Black population and 96 samples per subject for the White population.

Statistical calculations included values of bias (mean difference of SpO_2_-SaO_2_), precision (standard deviation [SD] of the difference), and accuracy (root-mean-square error [A_RMS_]). The distribution of the differences did not pass the Kolmogorov–Smirnov normality test, so a non-parametric Wilcoxon Rank Sum test was used to compare median values. Linear regression slope and intercept were calculated, along with the standard error estimate (SEE) for each group. In addition, the rate of occult hypoxemia (SaO_2_ < 88% when SpO_2_ = 92–96%) was determined for each self-declared race (Black and White).

## Results

**Fig. 2:**
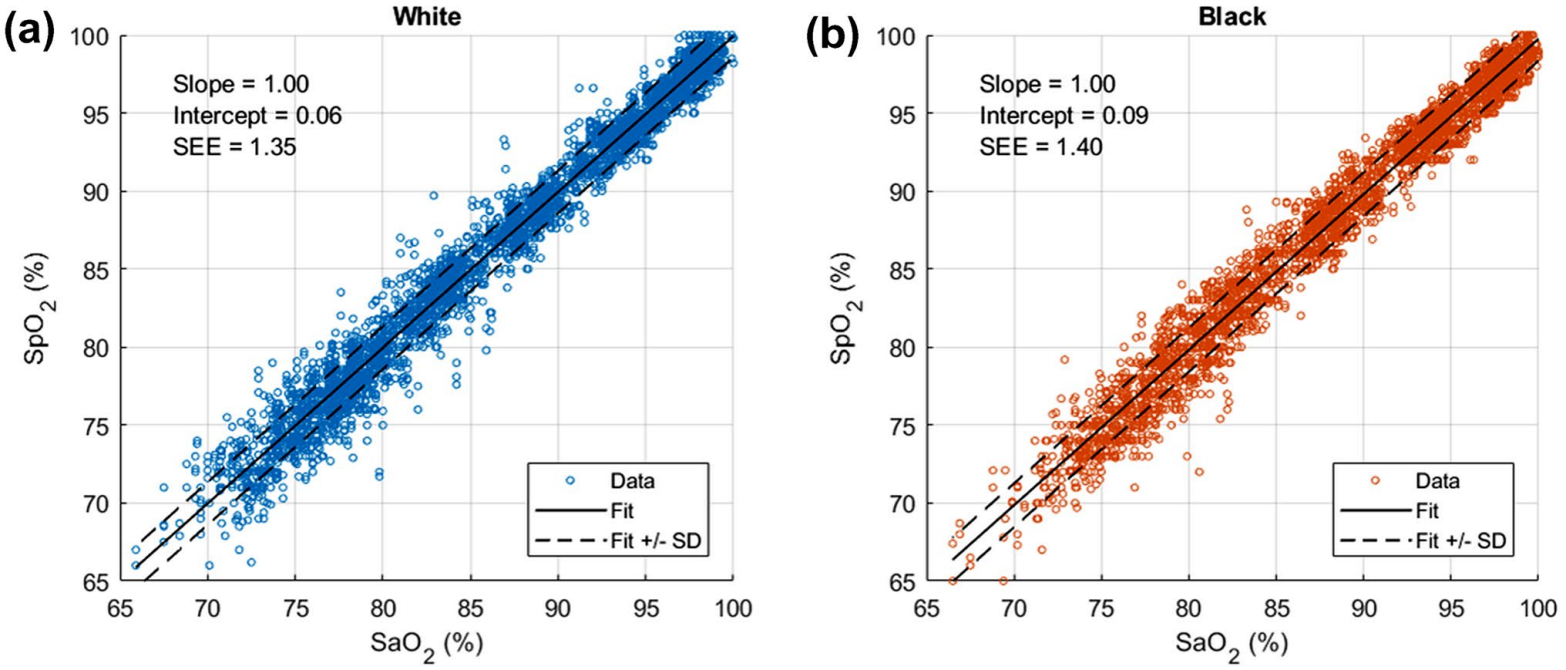
Scatter plot (SpO_2_ versus SaO_2_) along with performance metrics for White subjects (Fig. 2a) vs. Black subjects (Fig. 2b)

Figure 2 shows “scatterplots” of SpO_2_ versus CO-oximeter SaO_2_ for White and Black subjects. The solid lines show linear regression best-fits, and the dotted lines indicate the SEE limits. There are no significant visible differences between the two scatterplots. Statistical values for the two groups, including bias, precision, root-mean-square error, and numbers of data points are shown in Table 2. Bias and precision are − 0.2 ± 1.40% for Black subjects, and − 0.05 ± 1.35% for White subjects. Based on the Wilcoxon Rank Sum test, the median difference of − 0.2 was statistically significant (p < 0.001). All these error values are well within manufacturer accuracy specifications.

**Table 2:** Tabulated summary of performance statistics for Black, White and combined datasets

Masimo SET	Bias %	Precision %	A_RMS_ %	N_Pairs_	N_Subj_	Occult Hypoxemia%*
Black	− 0.20	1.40	1.42	3201	39	0
White	− 0.05	1.35	1.35	3982	36	0.2
All	− 0.12	1.37	1.38	7183	75	0.1

^*^Occult Hypoxemia = SaO_2_ < 88% when SpO_2_ = 92–96%

**Fig. 3:**
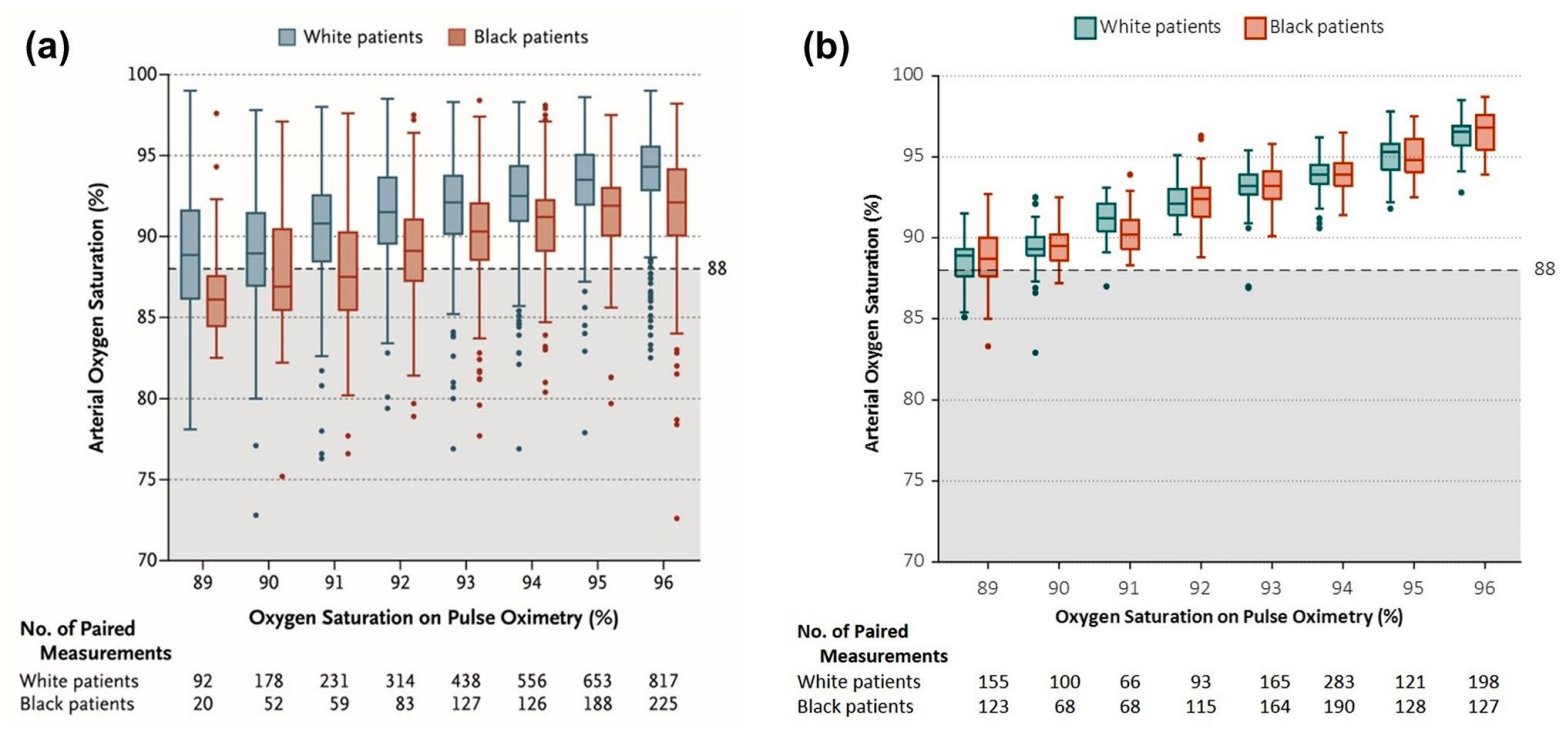
Box plots showing accuracy comparison for Black (salmon tint) and White (blue tint) ethnic groups binned by 1% saturation bins, from 89–96%. Figure 3a reproduced (with permission) from the Sjoding et al. study [12], and Fig. 3b similar box-plot configuration using Masimo SET® data pairs from the current laboratory study. Shaded area of SaO_2_%

To directly compare and contrast these data with the published results of Sjoding et al. [3], we plotted SaO_2_ versus SpO_2_ in the same manner as provided by those authors (Fig. 3). The box plot graphing protocol includes a horizontal line within each box representing the median; the top and bottom of each box represent the upper and lower limits of the interquartile range, and the whiskers represent 1.5 times the interquartile range. The Sjoding et al. data [3] (reproduced with permission) are depicted in Fig. 3a, whereas the Masimo data points from this study are plotted in Fig. 3b. Sjoding et al. state that true SaO_2_ values less than 88% combined with SpO_2_ values of 92–96% represent “occult hypoxemia” not detected by the pulse oximeter. Their plot (Fig. 3a) shows this to be more common in Black than White patients (reported as 11.7% and 3.6%, respectively). Our Masimo SET® data (Fig. 3b) do not demonstrate this tendency, as occult hypoxemia is calculated as 0% and 0.2% for Black and White subjects, respectively. The mean carboxyhemoglobin (COHb) was 1.1% for Black and 0.9% for White subjects, and mean Methemoglobin (MetHb) values were 1.1% for all subjects; COHb and MetHb values were < 1.9% for all subjects.

## Discussion

The data demonstrate clinically equivalent performance of Masimo SET® pulse oximeters with RD SET® sensors for healthy Black and White subjects. Due to the large number of paired samples a statistically significant difference was calculated between the biases (mean difference of SpO_2_-SaO_2_) obtained for Black and White subjects; however, this statistical finding is not relevant, as the numerical value is so small (0.15%, or approximately 1/7 of 1%) that it is not clinically significant. Furthermore, “occult hypoxemia,” as defined in the recent literature [3–7], did not occur in any Black subjects, and was present in only two data pairs from the White subject data pool. These two data pairs resulted in a 0.2% occult hypoxemia rate for White subjects. All accuracy and error values are well within manufacturer specifications.

Subjects who self-identified as Black had a Massey Scale median value of 6 (range 4–9), while those who self-identified as White had a Massey Scale median value of 2 (range 1–4), as shown in Table 1 and Fig. 1. Because individuals within a given race will have a range of skin pigmentation, it is expected that there will be a small number of individuals with similar levels of skin pigmentation in each race, as displayed in Fig. 1.

The absence of racial bias, and highly accurate overall performance exhibited by Masimo SET® pulse oximetry can be logically explained by Masimo’s engineering design and testing paradigm. When a Masimo SET® sensor is activated on a patient, the device adjusts the light intensity to optimize signal quality. This includes automatic adjustment of both light-emitting diode (LED) intensity and the receiver gain to compensate for differences in light absorbance. The light absorbance signal is then processed using Masimo’s “multiple parallel signal processing engine system” to calculate SpO_2_. Conventional pulse oximetry uses the standard red over infrared algorithm to provide SpO_2_, while Masimo SET® uses that conventional algorithm but has added four other algorithms that all run in parallel. These algorithms allow the distinction between arterial and venous signal during motion and low perfusion by identifying and isolating the non-arterial and venous noise SpO_2_ from the true arterial SpO_2_ components in the signal. These multiple signal processing engines work together to overcome limitations of each independent method. This advanced technique allows for a more accurate picture of the pulsatile (arterial) signal and significantly reduces the impact of static absorbers such as skin pigment and tissue thickness (e.g., finger, toe, or earlobe). Finally, the Masimo SET® SpO_2_ algorithm is calibrated and then validated using nearly equal numbers of dark and light-skinned subjects.

The recent retrospective studies analyzing the effect of skin pigmentation on pulse oximeter accuracy have had several common methodology limitations. First, most did not indicate pulse oximeter manufacturer, model, or sensor type used for data collection [3, 4, 6, 12–15], while the rare investigators who did list manufacturer(s), did not stratify their data by manufacturer [5–7]. Prior to this report, only two investigations studying racial differences in pulse oximetry identified the manufacturer(s) tested [16, 17]. It is important to emphasize that not all pulse oximeters are created equal with respect to skin color. In addition to significant technological differences, calibration and validation testing of pulse oximetry devices also vary between manufacturers. The U.S. FDA Guidance for medical-grade pulse oximeters requires a minimum of two subjects, or 15% of the study pool, to be dark-skinned during validation trials for 510(k) clearance. Masimo requires adherence to more rigorous pigmentation benchmark standards for calibration and validation studies, utilizing nearly equal numbers of dark-skinned and light-skinned subjects [8]. The rigor of this calibration and validation testing paradigm has helped ensure that Masimo SET® devices are accurate and reliable for all patients, regardless of race, ethnicity, or skin tone. This was demonstrated in a 2017 study comparing Masimo SET® (Radical-7®) pulse oximeters with another manufacturer’s devices in dark-skinned and light-skinned infants with hypoxemia [17]. These results showed an overall measurement bias of 0.8% for Masimo SET® compared to 3.9% for the other manufacturer’s device. Further, the bias difference between dark and light skinned infants was 1.4% for Masimo SET® compared to 2.4% for the other manufacturer’s device.

Other key methodological concerns with the recent retrospective clinical studies evaluating skin pigmentation and SpO_2_ accuracy include prolonged lag times between SpO_2_ and SaO_2_ readings, and absence of COHb and MetHb measurements. All of the recent studies allowed SpO_2_ measurements to be taken up to 5 min, or even 10 min of the SaO_2_ sample [3–7, 12, 13, 15, 16]. Per ISO 80601-2-61 standard, delays > 30 s are not considered current. It is widely recognized that blood oxygen saturation values can change significantly within this timeframe in patients [18]. In addition, most of these studies did not evaluate the impact of COHb and MetHb on SpO_2_ readings [4–7, 12, 14–16]. Elevated COHb and MetHb values are known to alter SpO_2_ values when measured using conventional pulse oximetry [19]. Furthermore, several of these studies evaluated pulse oximeter accuracy in COVID-19 patients [4, 6, 7, 16], and recent investigations demonstrate that COVID-19 patients can harbor significantly elevated endogenous COHb and MetHb values [20].

There are two notable limitations of our study. First, the retrospective data were collected from healthy subjects using a controlled laboratory desaturation protocol; thus, common clinical challenges to SpO_2_ readings present in critically ill patients (e.g., changes in breathing, anemia, abnormal extremity perfusion, etc.) were not present. However, this limitation should also be viewed as a strength of the study, as the elimination of known confounders facilitates greater focus on the evaluation of skin pigmentation. Furthermore, it should be recognized that desaturation studies can only be ethically performed on healthy subjects in a controlled laboratory setting. Second, this study is focused on the Black and White populations and does not evaluate other ethnic groups (i.e., Asian, Hispanic); however, this was done to maximize the contrast in skin pigmentation.

Future clinical studies on racial differences between SpO_2_-SaO_2_ measurements and the frequency of occult hypoxemia should examine critically ill patients using a prospective protocol that specifies the pulse oximeter manufacturer(s), model(s), and sensor(s). These studies should also include an objective measurement of skin pigmentation, such as the Massey Scale, to stratify the results by skin tone. In addition, SpO_2_ values should be recorded simultaneously with the SaO_2_ blood samples. Known pulse oximeter confounders, including elevated COHb and MetHb, should be excluded or evaluated in sub-group analyses. Finally, patient clinical status and drug history should also be recorded for sub-group analysis.

In conclusion, this retrospective study of healthy human volunteers monitored with Masimo RD SET® pulse oximeter sensors, showed an absence of clinically significant differences in accuracy between Black and White subjects. Furthermore, occult hypoxemia did not occur in Black subjects, and the occurrence of this finding was rare in White subjects. Prospective clinical studies are needed to validate these results in critically ill patients utilizing Masimo SET® pulse oximeters and other devices, stratified by manufacturer.

## Desaturation protocol exclusion criteria and summary of subject information

### Desaturation protocol exclusion criteria

- Subject < 18 years of age or > 50 years of age
- Subject weighs < 110 pounds
- Subject has a hemoglobin value < 11.0 g/dL
- Subject has Carboxyhemoglobin (COHb) value > 2.0%
- Subject’s baseline heart rate < 45 bpm or > 85 bpm
- Subject’s blood pressure
  - Systolic < 90 mmHg or > 140 mmHg
  - Diastolic < 50 mmHg or > 100 mmHg
- Subject is not able to read and communicate in English
- Subject does not understand the study and risks involved
- Subject is pregnant
- Subject has a body mass index (BMI in Kg/M^2^) > 35
- Subject has a history of fainting (vasovagal syncope), blacking out or losing consciousness during or after a blood draw, or has a fear of blood draws
- Subject has open wounds, inflamed tattoos or piercings, and/or has any visible healing wounds that a medical professional determines may place them at an increased risk for participation*
- Subject has known drug or alcohol abuse
- Subject uses recreational drugs*
- Subject experiences frequent or severe headaches and/or migraine headaches, migraine auras, altitude sickness, and/or headaches accompanied by visual changes or sensitivity to light or sound
- Subject has experienced a concussion or head injury with loss of consciousness within the past 12 months
- Subject has any history of a stroke, myocardial infarction (heart attack), and/or seizures
- Subject has any chronic bleeding disorder (e.g. hemophilia)
- Subject has taken anticoagulant medication within the past 30 days (excluding non-steroidal anti-inflammatory drugs (NSAIDS))
- Subject has donated blood within the past 4 weeks
- Subject has Wolff-Parkinson-White Syndrome or Stokes-Adams Syndrome
- Subject has any symptomatic cardiac dysrhythmia (e.g. atrial fibrillation) and has not received clearance from their physician to participate
- Subject has a known neurological and/or psychiatric disorder (e.g. schizophrenia, bipolar disorder, multiple sclerosis, Huntington’s disease) that interferes with the subject’s level of consciousness*
- Subject has taken opioid pain medication 24 h before the study
- Subject has any active signs and/or symptoms of infectious disease (e.g. hepatitis, HIV, tuberculosis, flu, malaria, measles, etc.)*
- Subject is taking medications known to treat any type of infectious disease
- Subject has either signs or history of peripheral ischemia or carpal tunnel syndrome
- Subject has had invasive surgery within the past year, including but not limited to major dental surgery, appendectomy, plastic surgery, jaw surgery, major ENT surgery, major abdominal and/or pelvic surgery, heart surgery, or thoracic surgery*
- Subject has symptoms of congestion, head cold, or other illness
- Subject has been in a severe car accident(s) or a similar type of accident(s) requiring hospitalization within the past 12 months
- Subject has any cancer or history of cancer (not including skin cancer)*
- Subject has chronic unresolved asthma, lung disease (including COPD) and/or respiratory disease
- Subject is allergic to lidocaine, chlorhexidine, latex, adhesives, or plastic
- Subject has a heart condition, insulin-dependent diabetes, or uncontrolled hypertension
- Subject has delivered vaginally, has had a pregnancy terminated, a miscarriage with hospitalization, or had a C-section within the past 6 months
- Subject intends on participating in any heavy lifting, repetitive movement of their wrist (including riding a motorcycle, tennis), exercise (working out, riding a bike, riding a skateboard, etc.), or any activity that will put additional stress on the wrist within 24 h following a study that involves an arterial line
- Subject has any medical condition which in the judgment of the investigator, and/or medical staff, renders them ineligible for participation in this study (Discretion of the investigator/study staff)

*Per physician discretion.

## Summary of subject information

- All subjects gave informed consent in person with a trained/delegated Clinical Lab staff member, on the day of study participation
- All subjects met inclusion criteria to participate
- Age range: 18–50
- BMI range: 19.6–33.1 (mean 26.4) (BMI calculated by NIH BMI calculator https://www.nhlbi.nih.gov/health/educational/lose_wt/BMI/bmicalc.htm)
- All subjects were non-smokers
- All subjects were not pregnant. All subjects with the ability to become pregnant were confirmed not pregnant via hCG urine test on the day of study participation.
- No subjects had chronic health conditions or diseases (e.g. cardiac issues, diabetes, hypertension, respiratory issues, cancer, etc.)
- Approximately 2/3 of subjects indicated they exercise regularly (1 or more times per week)
- One subject had an elective cesarean Sect. 10 months prior to study participation; the day of the study, the subject was healthy and was cleared to participate in the study at the discretion of the clinician. (Study criterion of 12 months following surgeries can be waived at the discretion of the physician).In addition, the C-section waiting requirement is 6 months post partum.
- One subject had a right-hand fracture 7 months prior to study participation; the physician deemed the subject eligible to participate, as the fracture was to the 5th metacarpal only, and was set without any hardware (plates/screws). The day of study participation, the subject had full range of motion, full strength, and no pain.
